# Depth resolved lattice-charge coupling in epitaxial BiFeO_3_ thin film

**DOI:** 10.1038/srep38724

**Published:** 2016-12-08

**Authors:** Hyeon Jun Lee, Sung Su Lee, Jeong Hun Kwak, Young-Min Kim, Hu Young Jeong, Albina Y. Borisevich, Su Yong Lee, Do Young Noh, Owoong Kwon, Yunseok Kim, Ji Young Jo

**Affiliations:** 1 School of Materials Science and Engineering, Gwangju Institute of Science and Technology, Gwangju 61005, Korea; 2 Department of Energy Science, Sungkyunkwan University (SKKU), Suwon 16419, Korea; 3 Center for Integrated Nanostructure Physics, Institute for Basic Science (IBS), Suwon 16419, Korea; 4 UNIST Central Research Facilities, Ulsan National Institute of Science and Technology, Ulsan 44919, Korea; 5 Materials Science and Technology Division, Oak Ridge National Laboratory, Oak Ridge, Tennessee 37831, United States; 6 Pohang Accelerator Laboratory, Pohang 37673, Korea; 7 Department of Physics and Photon Science, Gwangju Institute of Science and Technology, Gwangju 61005, Korea; 8 School of Advanced Materials Science and Engineering, Sungkyunkwan University, Suwon 16419, Korea

## Abstract

For epitaxial films, a critical thickness (*t*_c_) can create a phenomenological interface between a strained bottom layer and a relaxed top layer. Here, we present an experimental report of how the *t*_c_ in BiFeO_3_ thin films acts as a boundary to determine the crystalline phase, ferroelectricity, and piezoelectricity in 60 nm thick BiFeO_3_/SrRuO_3_/SrTiO_3_ substrate. We found larger Fe cation displacement of the relaxed layer than that of strained layer. In the time-resolved X-ray microdiffraction analyses, the piezoelectric response of the BiFeO_3_ film was resolved into a strained layer with an extremely low piezoelectric coefficient of 2.4 pm/V and a relaxed layer with a piezoelectric coefficient of 32 pm/V. The difference in the Fe displacements between the strained and relaxed layers is in good agreement with the differences in the piezoelectric coefficient due to the electromechanical coupling.

Interface of epitaxial complex oxide heterostructures determines functionalities such as ferroelectric polarization and piezoelectricity in a technological demand to reduce thickness of epitaxial thin films^1^,^2^,^3^,^4^,^5^. During the growth of epitaxial thin films, at a certain thickness, there can exist a boundary acting as an interface for lattice parameters as well as functionalities, so-called the critical thickness (*t*_c_)^6^,^7^,^8^. The layer below the *t*_c_, known as the strained layer, is strongly affected by the misfit strain posed by a substrate, including the clamping effect which is contrastable from bulk material. The layer grown above the *t*_c_, known as the relaxed layer, undergoes a structural phase transition that imposes a short range crystalline order with dislocation and crystalline mosaics (and/or flexoelectricity) which are directly linked to the functionalities of the thin film. However, both the structural phase and functionalities of the relaxed and strained layers in epitaxial thin films have not yet been determined due to the lack of experimental microscopies.

Among the complex oxides, ferroelectric thin films are suitable for use as a model system in which the effect of the thickness-dependent lattice parameters is critical for the representative functionalities, such as the remnant polarization and piezoelectricity. Experimental approaches for observing the ferroelectric response of strained layers to date have been focused on reducing the ferroelectric thin film thickness so that it is close to *t*_c_ [refs 8, 9, 10, 11, 12, 13], because conventional ferroelectric thin film analysis tools, such as piezoresponse force microscopy^14^ and laser scanning vibrometers^15^, only provide an average response that arises from both the highly strained and the relaxed layers. However, this experimental strategy suffers from some fundamental limitations, including an exponential increase in the leakage current^16^ and depolarization-field-induced degradation^17^. It is essential to use structural microscopy to resolve the individual ferroelectric responses of the strained layer and the relaxed layer in a ferroelectric epitaxial thin film.

In this paper, we report on the individual structural responses arising from the strained and relaxed layers of a ferroelectric BiFeO_3_ epitaxial thin film grown on SrRuO_3_/SrTiO_3_, including structural evolution, ferroelectricity, and piezoelectric responses. These were quantitatively investigated using scanning transmission electron microscopy (STEM) and time-resolved X-ray microdiffraction (TXRμD)^18^,^19^. In our experiments, we observed changes in the lattice parameters and displacement Fe cations associated with the ferroelectric polarization around 15 nm. We were able to differentiate the piezoelectric strain of the strained layer from that of the relaxed layer, which differed by one order of magnitude.

## Results and Discussion

**Figure 1:**
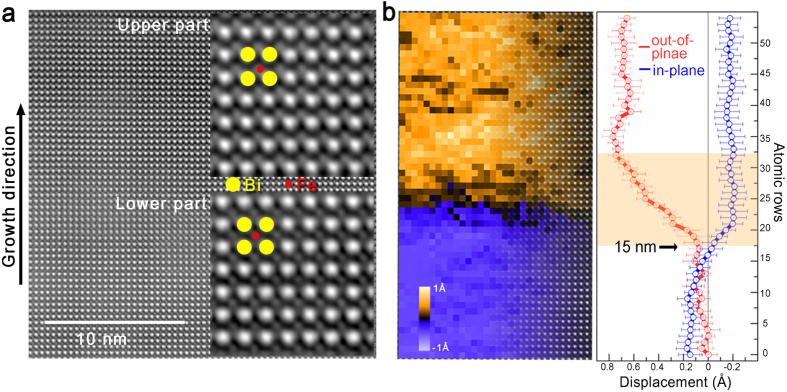
Structural transition associated with ferroelectric polarization at the critical thickness. (**a**) High angle annular dark-field (HAADF) images of BFO film on SRO/STO substrate. Insets in a show the magnified images extracted at the two regions, the upper and lower regions, separated by the boundary between the strained and relaxed layers. (**b**) Right: Out-of-plane displacement map of Fe cations in a BFO film showing the different polarization behaviour of the two regions of the upper and lower portions divided by the critical thickness boundary. Left: Corresponding averaged displacement profiles of the Fe cations along the in-plane and out-of-plane directions.

The theoretical *t*_c_ in epitaxial BiFeO_3_(BFO)/SrTiO_3_(STO) is estimated to be 14.9 nm using the formula developed Matthews and Blakeslee^20^, where the slip system of BFO is {101}<101>, the magnitude of the Burgers vector is 5.52 Å, and the angle between the dislocation line and the film/substrate interface is 45°. In order to directly observe the *t*_c_ in a BFO thin film, we selected a local area corresponding to a BFO thin film thickness of approximately 15 nm, as indicated in Fig. S1a. A high angle annular dark field (HAADF) STEM image of the BFO thin film on an SRO/STO substrate along the [001] pseudo-cubic direction shows an epitaxial thin film of high crystalline quality without defects (Fig. 1a and Fig. S1b). In contrast to experimental studies in epitaxial semiconducting films that report dislocation in layers above the *t*_c_ [ref. 21], defects, such as vacancies and dislocations, were not found in the BFO film near the theoretical *t*_c_. Instead, a change in the lattice parameters was observed along the growth direction using a magnified HAADF STEM image. The in-plane and out-of-plane lattice parameters for the upper part in Fig. 1a are 4.052 ± 0.089 Å and 3.928 ± 0.115 Å, while those for the lower part are 4.055 ± 0.091 Å and 3.906 ± 0.104 Å. This indicates that a BFO thin film possesses a relatively high *c/a* ratio of 1.038 below the theoretical *t*_c_. The small changes in the lattice parameters of the BFO thin film during growth is in good agreement with previous reports on the sustained lattice parameters for film thicknesses up to 100 nm^22^. However, it should be noted that the structural phase transition from higher symmetry to lower symmetry due to the generation of mosaics^23^ or oxygen octahedral tilting^2^, can be a possible mechanism for the change in lattice parameters around *t*_c_. In order to establish the relaxation mechanism in perovskite epitaxial films, further studies at the atomic scale are essential.

We found that the larger displacement of Fe cations from the center plane between the Bi sublattices in the upper portion, as shown in Fig. 1a, directly represents the larger ferroelectric polarization. The map for the out-of-plane displacement of Fe cations shown in Fig. 1b clearly shows a certain boundary thickness in order to distinguish the upper and the lower parts. In the upper region, which has a lesser degree of *c/a* ratio, the Fe cations were displaced toward the lower SRO electrode in comparison to that of the lower part, which indicates a higher polarization in the upward direction in a given field of view. The line profiles averaged over several atomic rows in the out-of-plane and in-plane directions also indicate that the boundary thickness where the change in the polarization magnitude begins is near atomic row number 17, which corresponds to a thickness of 15 nm. The displacements of the Fe cations in the upper and lower regions were approximately 0.7 Å and 0.1 Å with estimated error of ±0.1 Å as shown in Fig. S2, respectively. The value of approximately 0.1 Å in the lower part is similar to the value provided in previous reports for BFO thin films^24^, while the value of approximately 0.7 Å in the upper part is the largest value obtained so far. The large value that represents the cation displacement in the upper region can be attributed to the flexoelectric effect arising from the strain gradient near the *t*_c_ [refs 25,26] or small tilt effect due to sample bending in the upper part as compared to the lower part. It should be noted that when a thin film is subjected to strain gradient that varies in different localities, the local crystal orientation in the sample can even also accordingly change as shown in the FFT patterns for the lower and upper parts of BiFeO_3_ (Fig. S1). This effect might result in the deviation from the absolute magnitude of the Fe cation displacement to some small degree for the slightly-tilted part of BiFeO_3_ but should not be significant to the point of being misled in the estimation.

**Figure 2:**
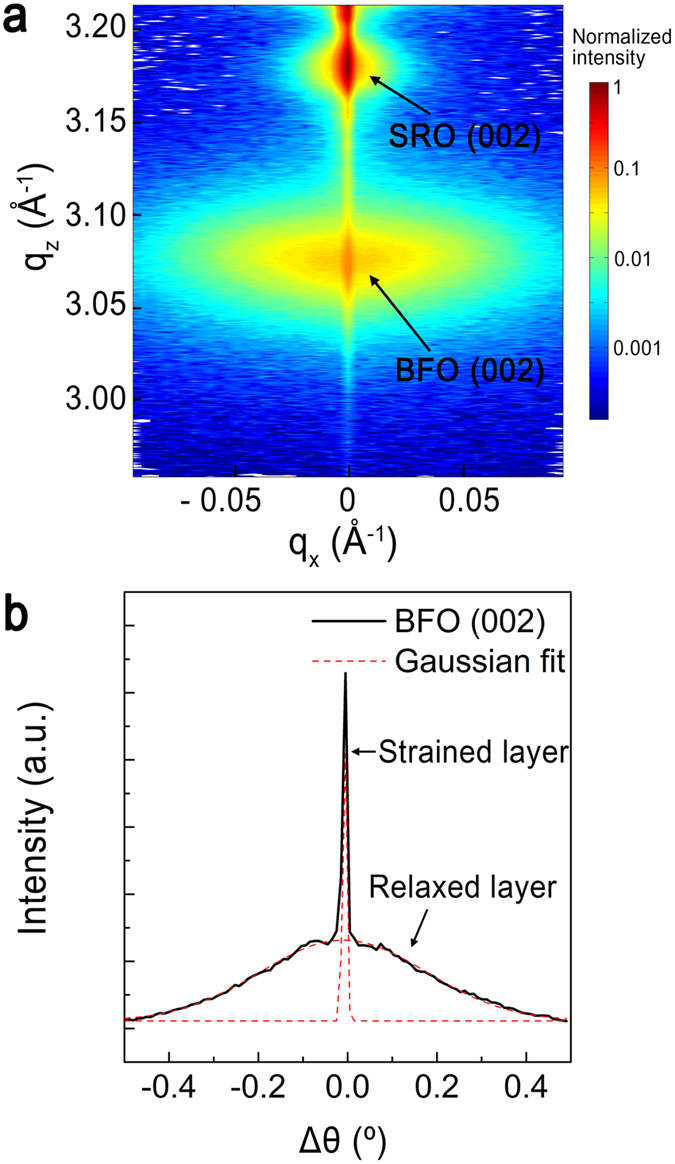
X-ray diffraction arising from two different layers. (**a**) RSM of an epitaxial BiFeO_3_ (002) thin film on SrRuO_3_ bottom electrode/SrTiO_3_ substrate. (**b**) (Black solid line): rocking curve of the (002) BiFeO_3_ reflection; (red dashed line): The result after fitting using two Gaussian distribution functions.

The regions that are above and below the *t*_c_ can be also assigned using the two peaks of the BFO (002) reflection with different full-width at half-maximum (FWHM) values in the *q*_z_ direction, as shown in Fig. 2a. The rocking curve of the BFO (002) reflection also consists of two peaks resulting from the strained and relaxed layers^23^,^27^ with different thicknesses and coherency, as shown in Fig. 2b. The narrower peak near the center of the rocking curve, which has a similar FWHM value to that of the STO substrate, is attributed to the strained layer arising from the lower part, whereas the broader peak can be attributed to the relaxed layer, which has a smaller differential orientation and short-range order. Based on the FWHM value along *q*_z_, the strained layer has a coherence length of 15 nm. This value is within 3% of the theoretical prediction for the *t*_c_ [ref. 7], where the angle between the dislocation line and the film/substrate interface is 45° [ref. 28].

**Figure 3:**
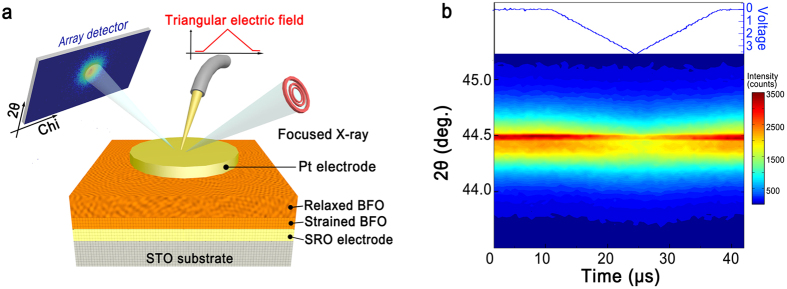
Time resolved X-ray microdiffraction (TXRμD). (**a**) Schematic of the TXRμD setup. (**b**) Time-resolved X-ray diffraction pattern of the BiFeO_3_ (002) reflection under an applied electric field. The top panel represents the temporal profile of the applied electric field.

In order to simultaneously probe the piezoelectric distortion of both the strained and relaxed layers, we performed TXRμD by applying a triangular electric field, as shown in Fig. 3a. Focused X-rays using a Fresnel zone plate were radiated onto the top electrode. The horizontal and vertical axes of the array detector screen indicate the chi and 2θ directions, respectively. The array detector was gated and delayed to measure the diffracted intensity before and after the beginning of the electric-field pulse. To confirm that there was contact between the probe tip and the top electrode, the output voltage generated by the BFO layer was also simultaneously measured.

We quantitatively investigated the piezoelectric distortion based on the changes in the 2θ value of the BFO (002) reflection under a 30 μs duration triangular electric wave, as shown in Fig. 3b. The 2θ peak for the strained layer was relatively narrow compared to the 2θ peak for the relaxed layer at a given scattering geometry, as shown in Fig. S4 in the Supplementary information. The diffraction peak for the relaxed layer shifts to a lower 2θ value under an electric field due to the increase in the lattice parameter *c* that arises from the piezoelectric expansion, whereas the diffraction peak with a higher intensity at 2θ = 44.493° that arises from the strained layer hardly shifts at all. The changes in the 2θ value and intensity that were caused by the presence of the electric field returned to their initial values when the electric field was turned off.

**Figure 4:**
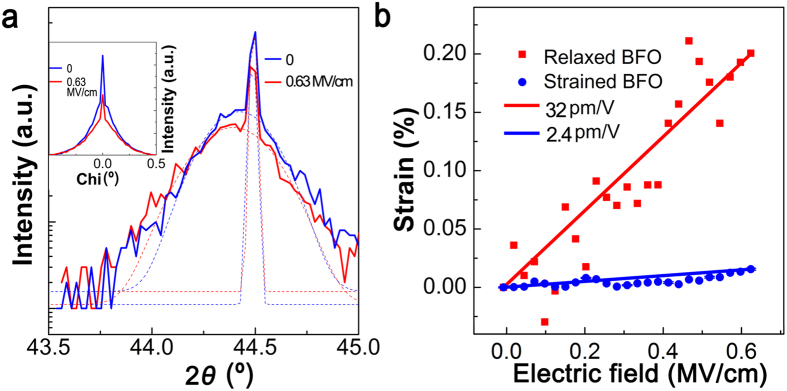
Piezoelectric behavior of the strained and relaxed layers. (**a**) Diffraction pattern as a function of 2θ for the (002) BiFeO_3_ Bragg reflection at (blue) *E* = 0 and (red) *E* = 0.63 MV/cm. The inset shows the diffraction pattern as a function of chi. (**b**) Piezoelectric strain of strained and relaxed layers as a function of *E*. The blue and green lines represent the linear fits.

The expansion of both the strained and relaxed layers was analyzed using the 2θ curves fit using two Gaussian distribution functions in an electric field (*E*) at 0 and 0.63 MV/cm, respectively (Fig. 4a). The diffraction peak for the strained layer was located at a higher 2θ value than that of the relaxed layer because the incident X-ray angle was lowered slightly to avoid an overlap of the diffraction peak centers from the strained and relaxed layers (in the Supplementary information). The center of the relaxed layer shifted from 2θ = 44.425° to 2θ = 44.398°, whereas that of the strained layer shifted from 2θ = 44.493° to 2θ = 44.486° at *E* = 0.63 MV/cm. The chi curve of the BFO (002) reflection, as shown in the inset of Fig. 4a, reveals no shift in the chi direction with the applied *E* for both the strained and relaxed layers, indicating that both layers have no distortion along the in-plane direction. The decrease in the peak intensity of the strained layer arises from the reduction induced by a decrease in the background signal due to a peak shift in the relaxed layer. The integrated intensity of the diffraction at *E* = 0 is equal to that at *E* = 0.63 MV/cm within 6%.

The piezoelectric strain-electric field curve was analyzed using the change in the peak position during the *E* pulse in a range from 0 to 0.63 MV/cm (Fig. 4b). The lattice parameter was converted using the 2θ value shown on the array detector screen, which can be expressed as a *q*_z_ value by projecting the cosine value of the difference between the incident X-ray angle and 2θ/2. The strain, using the normalization of the BFO lattice parameter at *E* = 0, is shown in Fig. 4b as a function of *E*. For the relaxed layer, the strain is proportional to *E*. The expansion of the relaxed layer at 0.63 MV/cm is approximately 0.2% with a piezoelectric coefficient of 32 pm/V. Our piezoelectric coefficient value for the relaxed layer is similar to the previously observed piezoelectric coefficient value of 40 pm/V for a pseudo–cubic 50 nm thick BFO film^29^,^30^,^31^. However, the estimated piezoelectric coefficient of the strained layer is 2.4 pm/V, which is much smaller than that of the relaxed layer (32 pm/V). Our piezoresponse force microscopy studies show that the BFO film exhibits a uniform piezoelectric response in an area of 5 × 5 μm^2^, indicating that the two different piezoelectric coefficients of the BFO may originate from the two types of layers along the thickness direction (Fig. S3 in Supplementary information). It is worthwhile to note that this difference between the piezoelectric coefficients (13 times) is a good match for the difference between the Fe displacements (7 times) for the strained and relaxed layers in a BFO thin film, where the piezoelectric coefficient is proportional to the polarization. Another possible reason for the suppressed piezoelectricity in the strained layer is the mechanical clamping effect for the strained layer posed by the substrate; however, further studies are required to determine if this is the case (see the Supplementary information).

The existence of a strained layer with an extremely low piezoelectric coefficient can lead to a strong thickness dependence in the piezoelectric coefficient, which is not yet in agreement with the theoretical calculations, as shown in Fig. S5. The piezoelectric coefficient of the strained layer can set a lower limit for the average piezoelectric coefficient of the epitaxial thin film, because the strained layer dominates the electromechanical behavior of ultrathin films. Using a combination of TEM and TXRμD studies, we were able to successfully resolve the structural evolution of strained and relaxed layers, thereby allowing us to avoid any extrinsic effects, such as the leakage current and depolarization field, and to describe a fundamental thickness limit for the piezoelectricity of ferroelectric ultrathin layers.

## Conclusion

In this paper, we resolved the ferroelectric and piezoelectric behaviors of both strained and relaxed layers in an epitaxial BFO thin film using a combination of TEM and TXRμD. We experimentally proved that the *t*_c_ is 15 nm for an epitaxial BiFeO_3_ thin film grown on SrRuO_3_/SrTiO_3_, which is in good agreement with the theoretical estimation. In addition to observing two different displacements of the Fe cation, we found that the strained layer below *t*_c_ exhibits an extremely low piezoelectric response (2.4 pm/V) in contrast to that for the relaxed layer (32 pm/V) due to the electromechanical coupling between the cation displacement and piezoelectric coefficient. From the point of view of strain engineering, the role of *t*_c_ was found to be critical in determining the unique functionalities of complex oxide thin films in contrast to those of bulk material.

## Methods

### Film growth

A 60 nm thick pseudo–cubic BFO layer with (001) orientation was prepared on fully strained SrRuO_3_ (SRO) on a (001)-oriented SrTiO_3_ (STO) substrate using pulsed laser deposition (PLD) with a pressure of 20 mTorr at 700 °C. We used a KrF excimer laser with a wavelength of *2*48 nm, an energy density of 1.5 J/cm^2^, and a repetition rate of 2 Hz, respectively. The 35 nm thick SRO layer was used as a bottom electrode. Top electrodes of with a diameter of 60 μm were deposited to form capacitor structures.

### Sample preparation and scanning transmission electron microscopy (STEM)

A cross-sectional sample for HAADF STEM imaging was prepared using a dual-beam focused ion beam (FIB, FEI Helios Nano Lab 450) slicing and lift-out technique and additionally thinned using a low-energy Ar-ion milling system (Fischione Model 1040 Nanomill) to remove any residual amorphous film. Atomic resolution HAADF STEM images of the sample were obtained using a 200 keV transmission electron microscope equipped with a probe-corrector (CEOS GmbH) and a cold field emission electron gun (JEM-ARM200F, JEOL Ltd., Japan). The angle range of the HAADF detector was from 75 to 175 mrad, and a probe formed a semi-angle of 20 mrad. The HAADF images used in the quantification of the atom positions to map out the Fe cation displacements inside the Bi lattice were denoised by a deconvolution of the probe function using a commercial software package (DECONV HAADF, HREM Research Ltd., Japan).

### X-ray diffraction analysis

Reciprocal space maps (RSMs) were analyzed to characterize the crystallographic structure of a BFO thin film a high-resolution X-ray diffractometer (Advanced D8, Bruker). The piezoelectric strain was investigated using TXRμD at the 9 C beamline at the Pohang Accelerator Laboratory. Monochromatic X-rays with an energy of 8 keV were focused on a 10 μm spot size using a Fresnel zone plate. To both apply the electric field and simultaneously check the ferroelectric signal, two customized probes were attached on the x–y stage of a goniometer for electrical contact to the top and bottom electrodes during the TXRμD experiments. A gated array detector (Pilatus 100 K, Dectris) collected the diffracted intensity in real space. In order to avoid electrical damage to the BFO capacitors from the number of applied electric pulses, we reduced the number of repetitions of the electric pulse by fixing both the incident X-ray angle and the position of the array detector.

## Additional Information

**How to cite this article:** Lee, H. J. *et al*. Depth resolved lattice-charge coupling in epitaxial BiFeO_3_ thin film. *Sci. Rep.* **6**, 38724; doi: 10.1038/srep38724 (2016).

**Publisher's note:** Springer Nature remains neutral with regard to jurisdictional claims in published maps and institutional affiliations.

## Supplementary Material

Supplementary Information
