# Supplementary material for: Depth resolved lattice-charge coupling in epitaxial BiFeO3 thin film
Source: Sci Rep. 2016 Dec 8;6:38724. doi: 10.1038/srep38724 (PMC5144002; doi:10.1038/srep38724)
Supplement: Supplementary Information [file srep38724-s1.pdf]

Supplementary information

Depth resolved lattice-charge coupling in epitaxial $BiFeO_3$ thin film

Hyeon Jun Lee[1], Sung Su Lee[1], Jeong Hun Kwak[1],Young-Min Kim[2,3], Hu Young Jeong[4], Albina Y. Borisevich[5], Su Yong Lee[6], Do Young Noh[7], Owoong Kwon[8], Yunseok Kim[8] & Ji Young Jo[1,*]

[1]School of Materials Science and Engineering, Gwangju Institute of Science and Technology, Gwangju 61005, Korea.

[2]Department of Energy Science, Sungkyunkwan University, Suwon 16419, Korea.

[3]IBS Center for Integrated Nanostructure Physics (CINAP), Institute for Basic Science, Sungkyunkwan University, Suwon 16419, Korea.

[4]UNIST Central Research Facilities, Ulsan National Institute of Science and Technology, Ulsan 44919, Korea.

[5]Materials Science and Technology Division, Oak Ridge National Laboratory, Oak Ridge, Tennessee 37831, United States.

[6]Pohang Accelerator Laboratory, Pohang 37673, Korea.

[7]Department of Physics and Photon Science, Gwangju Institute of Science and Technology, Gwangju 61005, Korea.

[8]School of Advanced Materials Science and Engineering, Sungkyunkwan University, Suwon 16419, Korea.

*Correspondence and requests for materials should be addressed to J.Y.J. (e-mail: jyjo@gist.ac.kr).

1. Cross sectional TEM

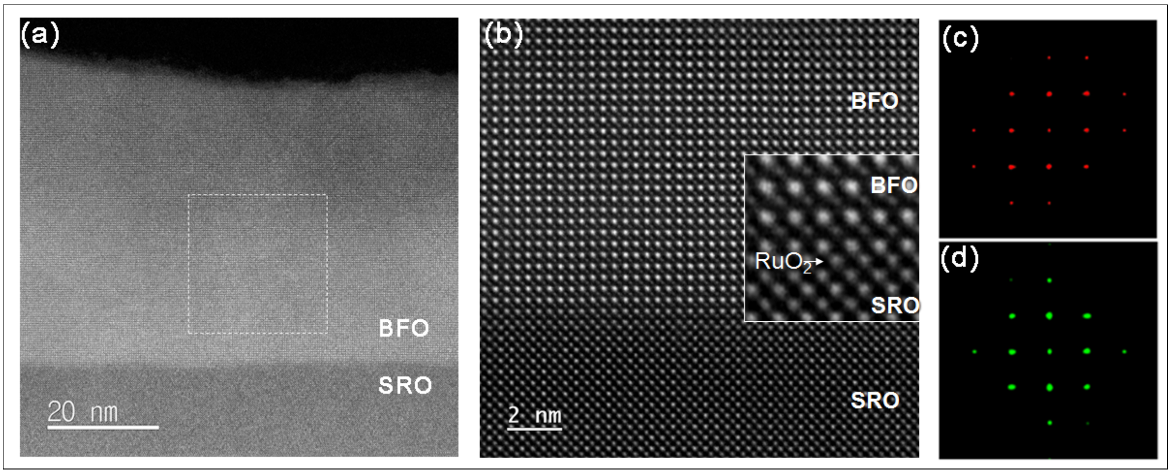

Figure S1. (a) Cross-section images of the BFO/SRO film. (b) High angle annular dark-field (HAADF) images of the BFO film near the interface. Insets in (b) show magnified images extracted near the interface of the BFO/SRO film, indicating that epitaxial film of high quality show a clear interface between the BFO and SRO. Significant changes in the Fe displacement were not found below 15 nm of thickness of the BFO film. (c,d) Fast Furier Transform (FFT) images of upper and lower part as shown in Figure 1a.

To estimate statistical variation of the measurements of Fe ion displacements in the BFO film, the HAADF STEM image of $SrTiO_3$ substrate taken at the same imaging condition was used as a standard. We averaged measured spacing of the lattice parameters and Ti (B-site) ion displacements along the out-of-plane and in-plane directions as shown in Supporting Figure S2b and c. Consequently, the averaged measured spacing in this method can vary with the measurement error of ~± 11 pm. It has been demonstrated that our computational approach can measure position shifts of atomic columns with sub-5 pm precision on a HAADF STEM image acquired at optimal imaging condition [*Nat. Mater.* **11**, 888-894 (2012)].

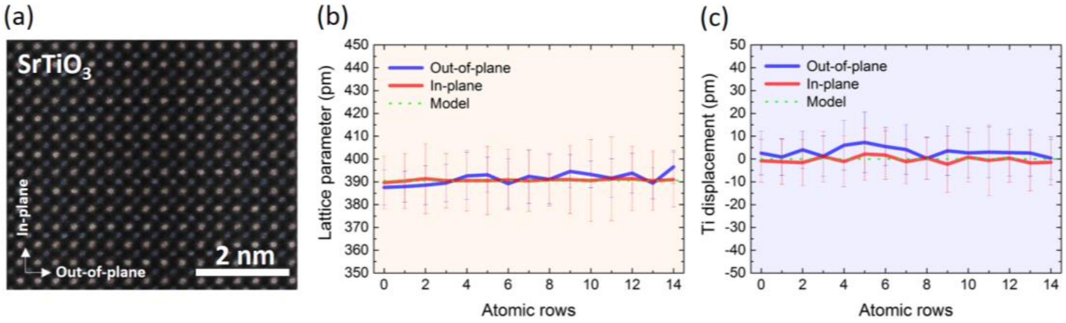

(d)

Measurement error for Lattice parameter		Measurement error for Ti displacement	
Out-of-plane	In-plane	Out-of-plane	In-plane
± 8.47 pm	± 13.84 pm	± 9.99 pm	± 10.94 pm
Averaged measurement error = ± 10.81 pm			

Figure S2. Measurement error of the atom position mapping. (a) The result of peak findings in the HAADF STEM image of $SrTiO_3$ substrate by tracking the highest intensities of the constituent bright atom positions with computational iterations. (b, c) Profiles of the measured lattice parameters and Ti displacements averaged over the vertical atomic rows of the HAADF STEM image. The error bars in each graph show the standard deviations

2. PFM study

The topography and ferroelectric information were simultaneously studied using piezoresponse force microscopy (PFM) with 17 kHz of 1 V_{ac}. As shown in Fig. S3 (a), the surface of the BFO/SRO/STO consists of a number of particles caused by relaxation during the growth of the BFO film. In Fig. S3 (b), the red and blue solid boxes were applied using +6 V_{dc} and –6 V_{dc} for switching the ferroelectric domains. The red solid box shows that it is possible to achieve uniform ferroelectric switching and amplitude magnitude even in the presence of particles.

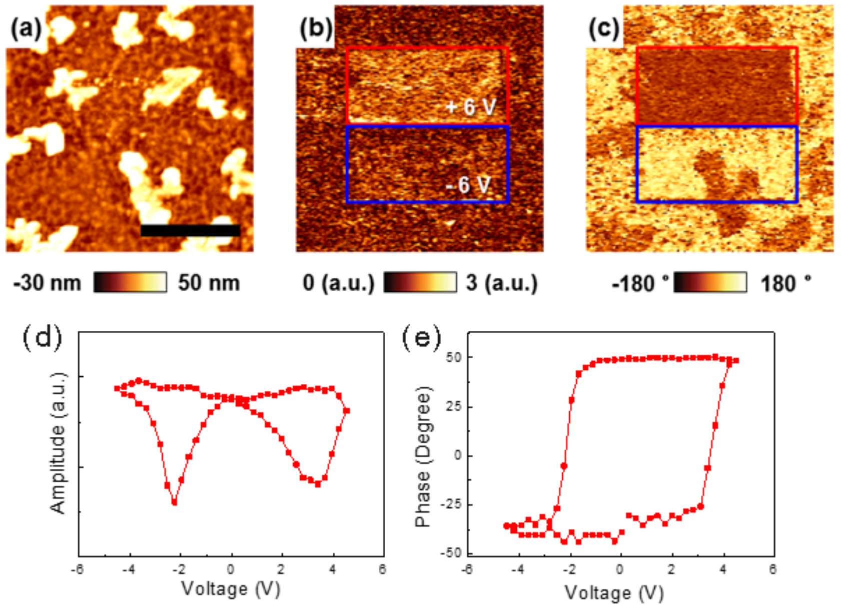

Figure S3. (a) Topography and PFM (b) amplitude and (c) phase images of the BFO/SRO/STO. Scale bar is 2 μm. (d) amplitude and (e) phase curves.

3. An array detector screen in reciprocal space

When an X-ray is used to irradiate an array detector screen with an incident angle and specific energy, wave vector k_i is used to represent an incident X-ray for a line in reciprocal space, as shown in Fig. S4 (a). Compared to a point detector, an array detector screen possesses a wide 2θ range and catches scattered X-rays that have various scattering angles. Because the scattering vector Q is a difference vector between k_i and k_f, a group of scattering vectors Q at the array detector is inclined in the q_y direction.

Inclined scattering vectors of the array detector cross (002) the reflection of both the strained and relaxed BFO simultaneously without moving the motor of the X-ray diffractometer. Due to the broad scattering vector of the relaxed BFO along with q_y, the

diffraction peak of the relaxed BFO is wider than that of the strained BFO, as shown in Fig. S4 (b).

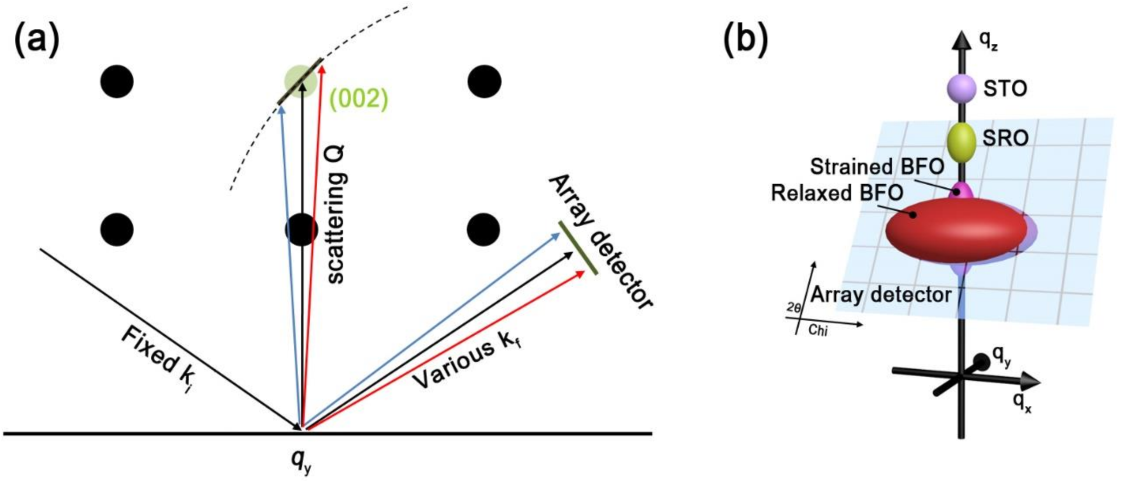

Figure S4. Schematic for (a) Incline reciprocal space with various scattering angles arising from an array detector screen, and (b) crossing the screen of an array detector towards both the strained and relaxed layers in reciprocal space.

4. Influence of a highly strained layer on the average d_{33}

The influence of a highly strain layer on the average $d_{eff,33}$ value and thickness direction of the thin film can be expressed by

$$d_{eff,33} = \frac{d_c \times t_c + d_r \times t_r}{t}, \quad (1)$$

where d_c, d_r, t_c, t_r, and t are the d_{33} of the strained layer, d_{33} of the relaxed layer, the thickness of the strained layer, and the thickness of the relaxed layer, respectively. In the case of BFO/SRO/STO, the measured d_c and t_c was 2.4 pm/V and 15 nm, respectively. The dashed line in Fig. S2 shows the ratio between the calculated $d_{eff,33}$ using Eq.(1) and the d_{33} of bulk

BFO. When compared to the previous results, the existence of the highly strained layer may be the reason for the abrupt reduction in the d_{33} value when the thickness is decreased below 60 nm.

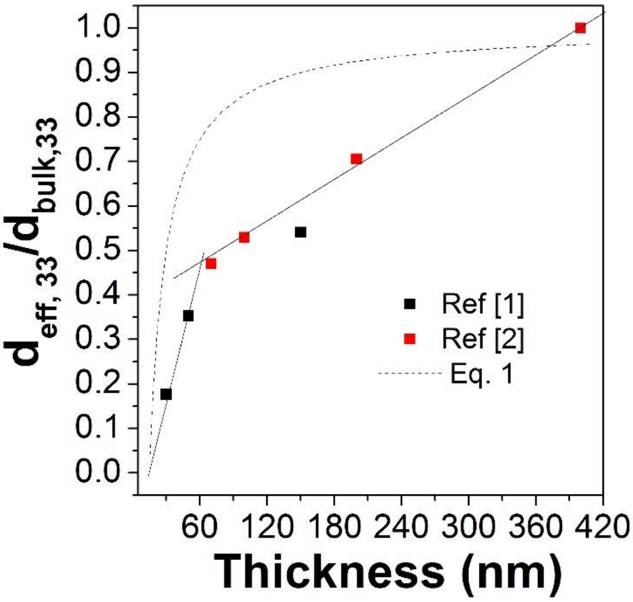

Figure S5. The ratio of the effective piezoelectric coefficient of BFO film to that of bulk material. The dashed line was calculated using Eq. (1). The black and red squares were reproduced from Ref. [1] and Ref [2], respectively.

The differences in the piezoelectric coefficients can be explained by the relationship between the compressive stress, which arises from the misfit strain between the substrate and film, and the film thickness based on the formula $\sigma_0 \exp(-kz)$], where σ_0, k, and z are the maximum stress, decline parameter, and thickness, respectively[20,31]. Whereas the stress gradually decreases with the increase in the film thickness for a film region above t_c, the stress in the highly strained layer is consistent. When the value of k for ferroelectric film is

approximately 0.004/nm, the misfit strain between the STO substrate and BFO material can impose as much as a 5 times higher compressive stress to the highly strained layer below the t_c than that of the relaxed BFO at a thickness of 60 nm, thereby indicating a suppressed piezoelectric expansion in the strained layer. With an increase in the thickness of the film, we can expect an increase in the piezoelectricity of the relaxed layer, while the piezoelectricity of the highly strained layer becomes suppressed. In addition, the higher piezoelectric coefficient of the relaxed layer could originate from the mosaicity due to the structural relaxation behavior of the BFO film above t_c.

Ref [1]: Zhao, J. L., Lu, H. X., Sun, J. R. & Shen, B. G. Thickness dependence of piezoelectric property of ultrathin $BiFeO_3$ films. *Physica B: Condensed Matter* **407**, 2258-2261.

Ref [2]: Wang, J. *et al.* Epitaxial $BiFeO_3$ Multiferroic Thin Film Heterostructures. *Science* **299**, 1719-1722.

5. Evolution of rocking curve with various thickness

By increasing thickness of $BiFeO_3$ film, the rocking curve of the BFO (001) gradually consisted of narrow and broad peaks. The intensity of narrow peak arising from strain layer was also conserved at 65 nm thick film. We think that this results obviously indicate narrow peak and broad peak are originated from strained layer and relaxed layer, respectively. Strain relaxation behavior along the growth direction can make different structural features.

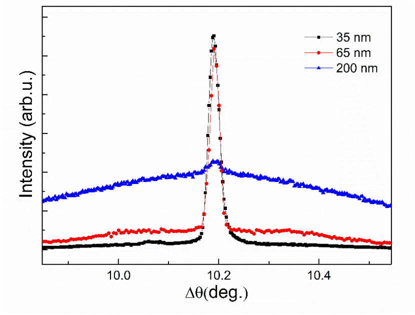

Figure S6. XRD rocking curves with various thickness of BiFeO3 thin films on SRO/STO substrate.

At much thicker film, we found that narrow peak from strained layer almost disappeared. It indicates that increasing thickness can influence the strained layer near interface of heterostructure. So, we think that it will be difficult to find a distinct boundary for strained and relaxation layer at thicker film. The integrated intensity of 200 nm-thick $BiFeO_3$ in rocking curve is 11 times higher than 35 nm-thick $BiFeO_3$ film.
